# Non-Autogenous Innovative Reconstruction Method Following Mandibulectomy

**DOI:** 10.3390/medicina56070326

**Published:** 2020-06-30

**Authors:** Bahaa Haj Yahya, Eli Rosenfeld, Gavriel Chaushu, Ilana Kaplan, Yehonantan Ben-Zvi, Yafit Hamzani

**Affiliations:** 1 Department of Oral and Maxillofacial Surgery, Rabin Medical Center—Beilinson Hospital, Petach Tikva 4941492, Israel; yafitha@clalit.org.il (B.H.Y.); eliros@gmail.com (E.R.); yonident@gmail.com (Y.B.-Z.); 2 Department of Oral and Maxillofacial Surgery, The Maurice and Gabriela Goldschleger School of Dental Medicine, Tel Aviv University, Tel Aviv; Head, Department of Oral and Maxillofacial Surgery, Rabin Medical Center—Beilinson Hospital, Petach Tikva 4941492, Israel; gabi.chaushu@gmail.com; 3 Department of Oral Pathology, Rabin Medical Center—Beilinson Hospital, Petach Tikva 4941492, Israel; Dr.ilanakaplan@gmail.com

**Keywords:** ameloblastoma, mandible, bone block, allogenic, autogenic, rhBMP

## Abstract

Plexiform ameloblastoma is a locally aggressive odontogenic tumor, rare in the anterior mandible. The treatment of choice is resection with 1–3 cm free margins. In most of reported cases, the affected mandible is reconstructed by autogenic bone graft or osseocutaneous microvascular free flap in order to return function and esthetics. Case description: A 2 cm diameter exophytic ameloblastoma, located in the anterior mandible of a 50-year-old male was resected and reconstructed in a unique manner—allogenic bone block, recombinant human bone morphogenetic protein (rhBMP) and xenograft particles via transcutaneous submental approach. After bone maturation, dental implants were placed and restored by fixed prosthetics. Practical implications: Mandible reconstruction modalities have a crucial influence on patient quality of life, function and esthetics. Allogenic bone block combined with rhBMP and xenograft particles can replace the traditional autogenous bone in certain circumstances. A submental transcutaneous “tent pole” approach can improve the success rate of the reconstruction procedure.

## 1. Introduction

Ameloblastoma, a benign tumor of odontogenic epithelial origin, occurs in 0.78% of oral cavity neoplasia [1,2]. For the plexiform type, radical resection with a safety margins of 1 cm to 3 cm is recommended [1,3]. Resection in the mandible can lead to either marginal or segmental mandibulectomy defects. Marginal mandibulectomy defects involve the resection of a single cortex of the mandible and can be restored in different ways, such as osseocutaneous microvascular free flaps or non-vascularized bone grafts. The most popular among the former is fibula due to its long pedicle, ease of contouring with multiple osteotomies, and suitability as a recipient site for endosseous implants [4].

Autogenous bone is considered the “gold standard” in grafting surgeries due to its properties of osteogenesis and osteoinduction [5,6]. However, it poses disadvantages such as high resorption rates, morbidity of the harvesting site and limited availability of intra-oral sources [6]. Other sources include allogeneic, alloplastic, and xenogeneic materials [7].

Allogeneic bone may be available in many forms, such as cancellous, corticocancellous, cortical graft, osteochondral, whole bone segment, and demineralized bone matrix. The graft integration process of allogeneic bone is similar to that which non-vascularized autogenous bone graft generally undertakes, but the size of the allograft influences the time of incorporation [8].

A transcutaneous submental approach was suggested for reconstruction of severely resorbed mandibles, where 4 to 6 dental implants were placed simultaneously with the bone graft to act as “tent poles” to maintain the height of the overlying mucosal soft tissue [9].

The aim of this article is to present a treatment workflow of mandible plexiform ameloblastoma with a unique reconstruction method, that can, in certain circumstances, replace the traditional one. The rationale of the study was to prevent side effects and complications following autogenic bone graft or osseocutaneous microvascular free flaps. The null hypothesis was that the non-autogenous reconstruction method can be used as an alternative treatment option in cases similar to the one presented.

## 2. Case Presentation

**Figure 1:**
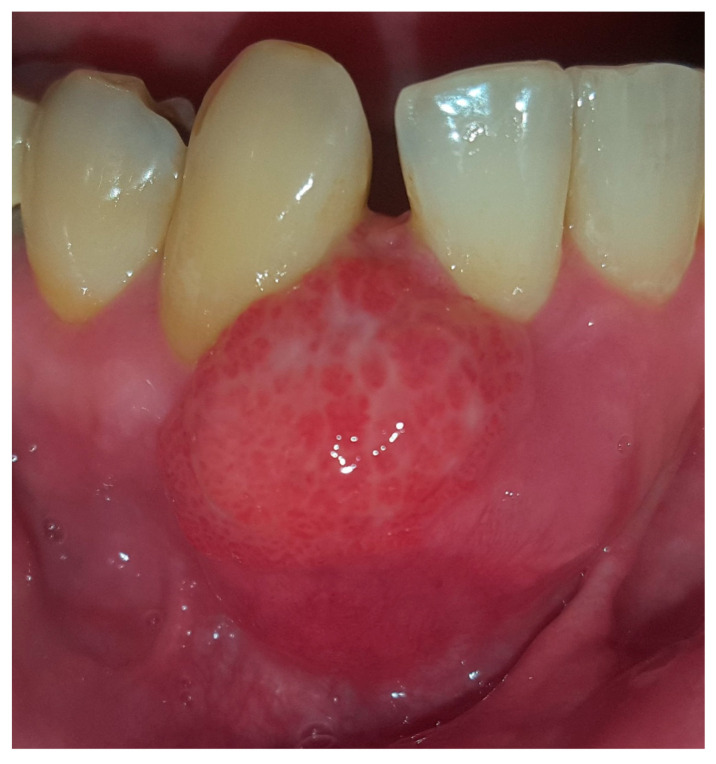
Reddish with white stripes exophytic 2 cm diameter mass located buccal to right anterior mandibular teeth.

**Figure 2:**
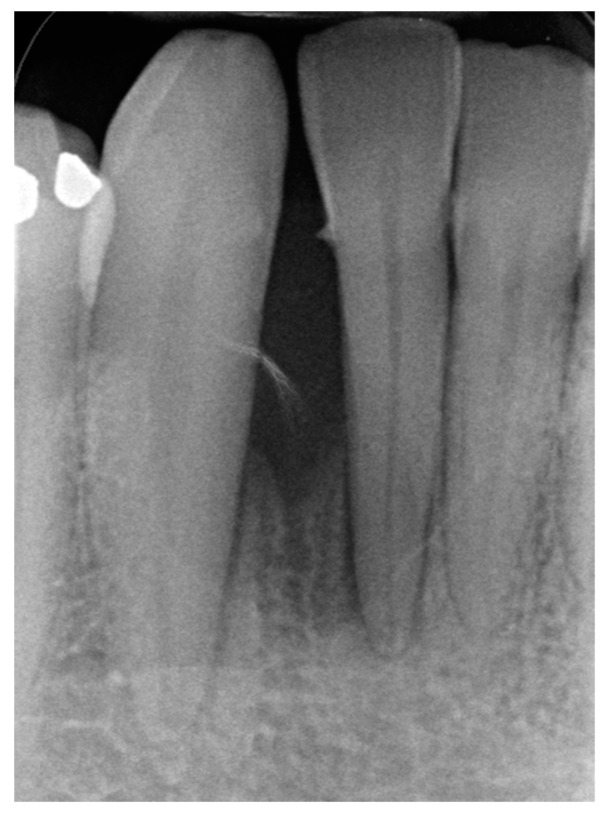
Resorption of alveolar bone surrounding and root deviation of lateral incisors as evident in dental X-ray.

**Figure 3:**
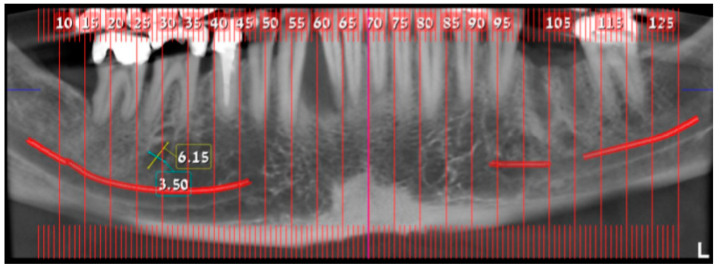
Resorption of alveolar bone surrounding and root deviation of lateral incisors as evident in CBCT (cone beam computed tomography, mentioned in the text above); panoramic image of reconstruction.

**Figure 4:**
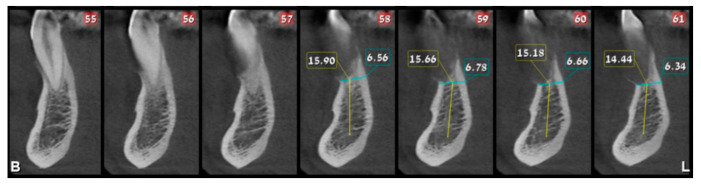

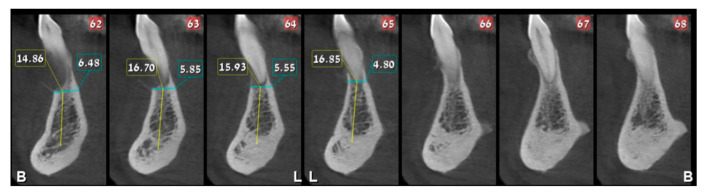
Resorption of alveolar bone surrounding and root deviation of lateral incisors as evident in CBCT; cross-sections of reconstruction.

**Figure 5:**
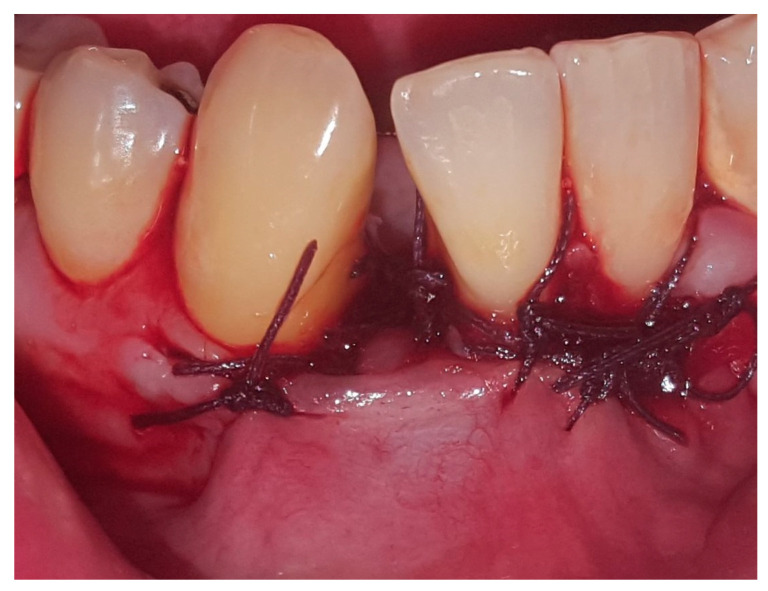
Clinical appearance following incisional biopsy and soft tissue suturing by 3-0 Vicryl.

**Figure 6:**
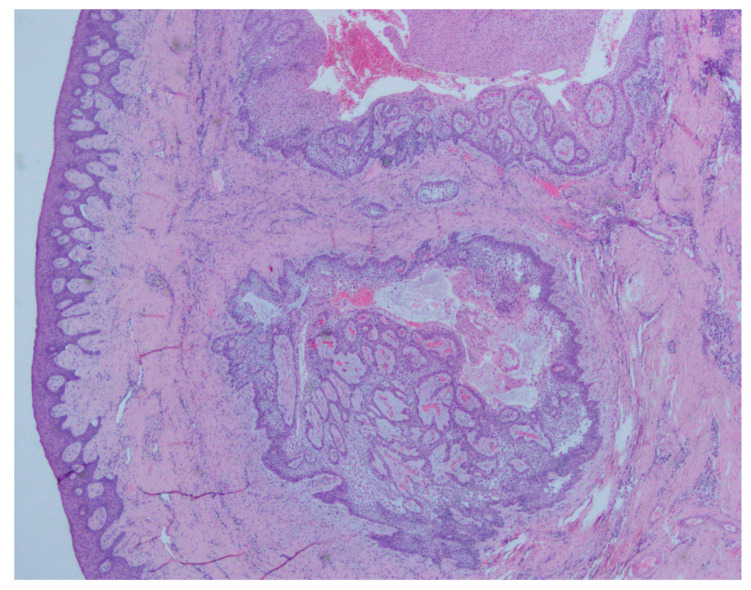
At low magnification, exhibiting islands of ameloblastoma in the sub-mucosa, with a plexiform architecture in a dense matrix (H&E original magnification × 40).

**Figure 7:**
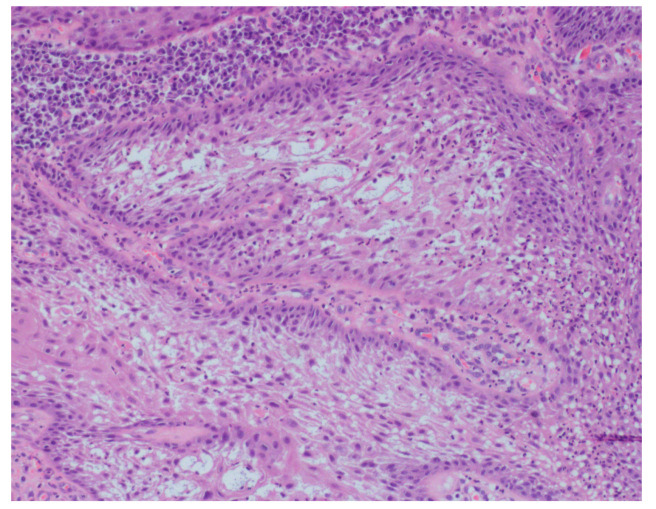
Polarization of the basal nuclei and stellate reticulum-like appearance of the cells in the central zone are demonstrated. (H&E, original magnification × 200).

In August 2017, a 48-year-old male, was referred to Rabin Medical Centre for the evaluation of a 2 cm diameter exophytic mass located buccally to right anterior mandibular teeth (Figure 1). The patient is a former smoker (quit five years before referral time) and has diabetes mellitus, hyperlipidemia and oral sleep apnea. Resorption of alveolar bone and root deviation of the right lateral incisor was evident in dental X-ray (Figure 2) and in cone beam computed tomography (CBCT) imaging (Figure 3 and Figure 4). In clinical examination, the tooth was vital and mobile class 3 according to Miller Classification. Incisional biopsy of the lesion was done (Figure 5) and revealed a plexiform ameloblastoma (Figure 6 and Figure 7). Microscopically, a solid epithelial tumor was observed composed of interdigitating islands of odontogenic epithelium, with stellate reticulum-like areas, polarization of the basal nuclei and a dense matrix. The tumor cells were bland, lacking any sign of atypia. The final diagnosis was plexiform ameloblastoma.

**Figure 8:**
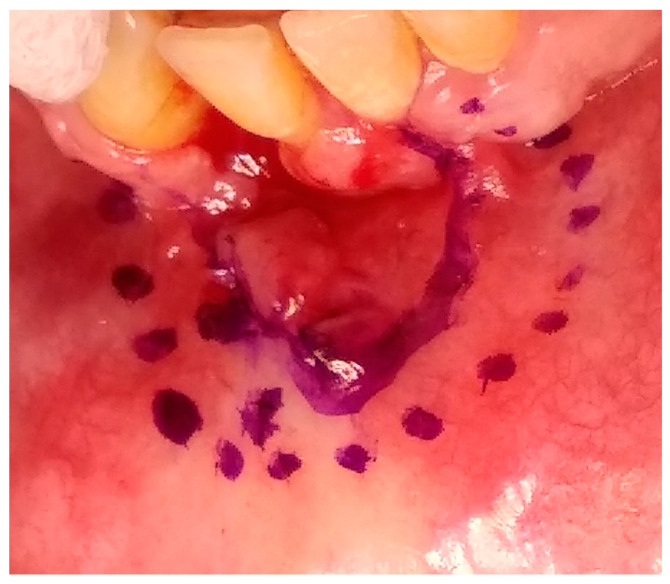
Tumor resection with 1 cm margins, soft tissue marking.

**Figure 9:**
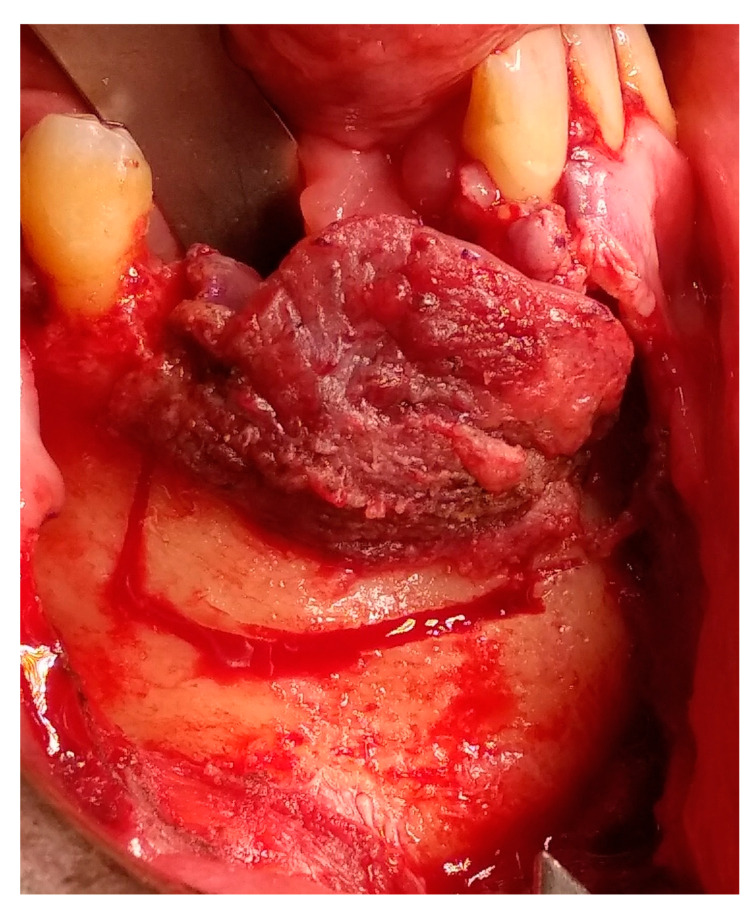
Tumor resection with 1 cm margins, osteotomy marking.

**Figure 10:**
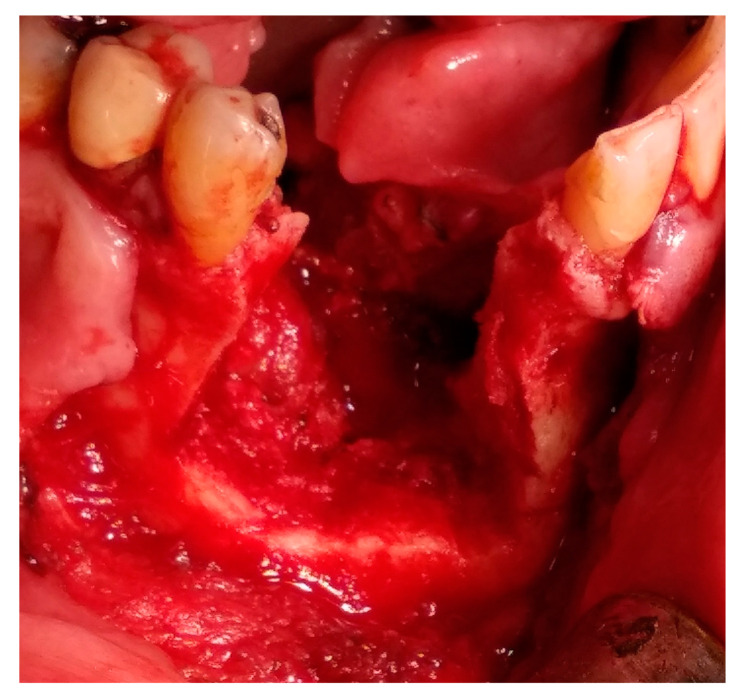
Tumor resection with 1 cm margins, resulting in marginal mandibulectomy.

**Figure 11:**
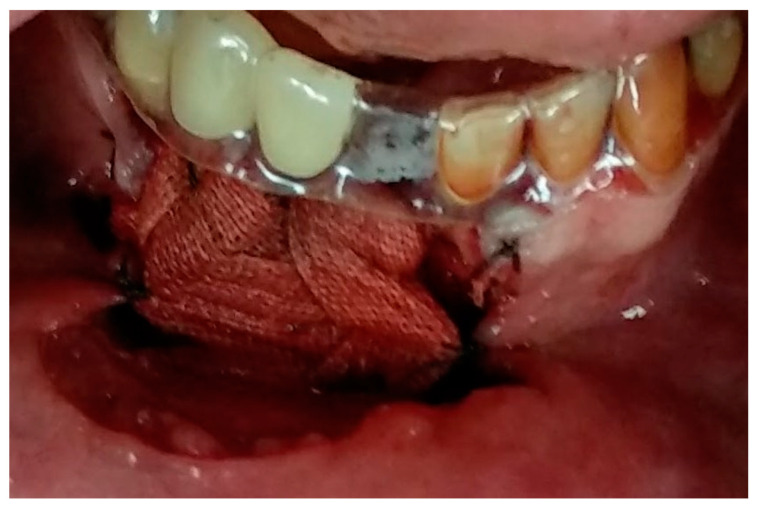
Essix splint on adjacent teeth and gauze packing in wound bed.

**Figure 12:**
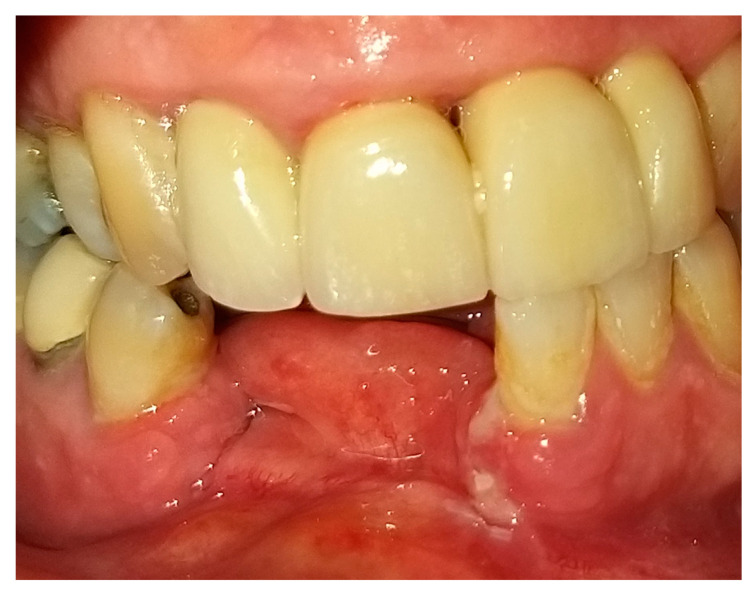
Clinical appearance of vertical defect in anterior mandibular alveolar ridge.

**Figure 13:**
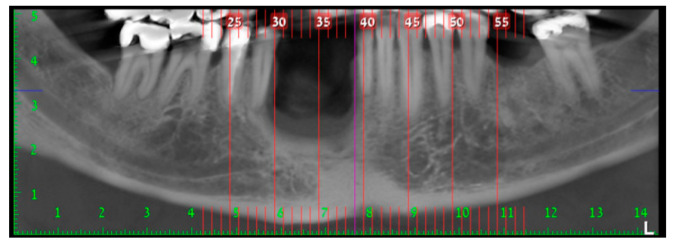
Two cm vertical defect in anterior mandibular alveolar ridge in CBCT imaging; panoramic image of reconstruction.

**Figure 14:**
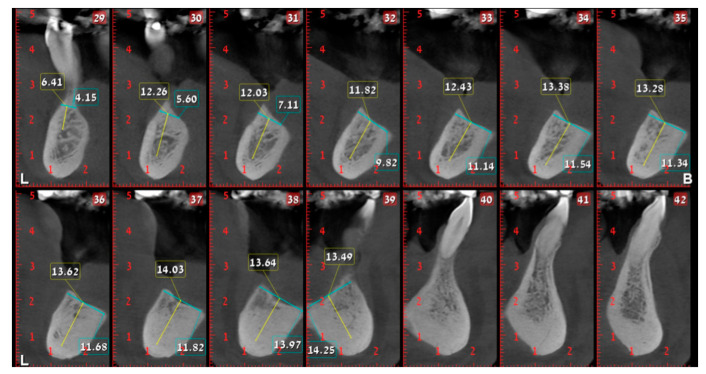
Two cm vertical defect in anterior mandibular alveolar ridge in CBCT imaging; cross-sections of reconstruction.

Having a finite diagnosis and under general anesthesia, the mass was resected with 1 cm free margins, resulting in marginal mandibulectomy (Figure 8, Figure 9 and Figure 10). Following the resection, the patient received an Essix splint and gauze packing in order to allow secondary healing (Figure 11). Post-operative recovery was fair, with normal inferior alveolar nerve function and no evidence of recurrence during two years follow-up. A vertical defect of 2 cm in the anterior mandibular alveolar ridge was stable during the follow-up period as could be seen clinically (Figure 12) and by CBCT (Figure 13 and Figure 14).

**Figure 15:**
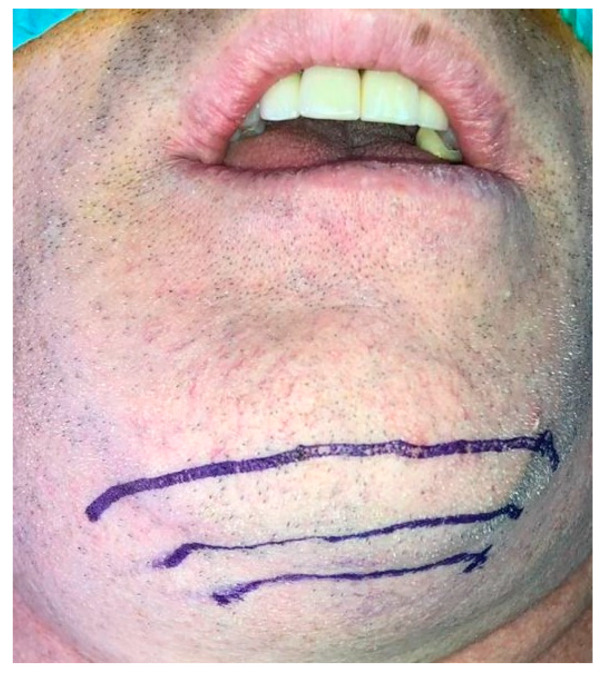
Submental transcutaneous tent pole approach markings.

**Figure 16:**
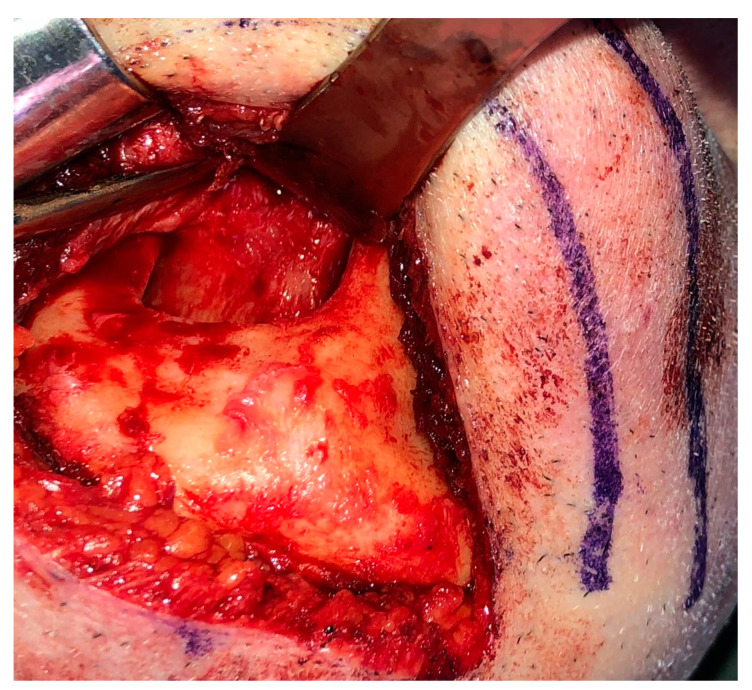
Defect exposure throughout submental transcutaneous tent pole approach.

**Figure 17:**
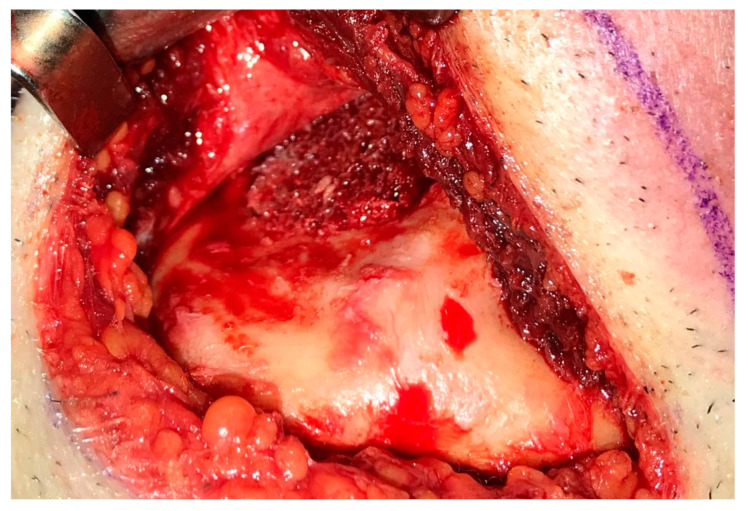
Allogenic bone block is fitted to the defect.

**Figure 18:**
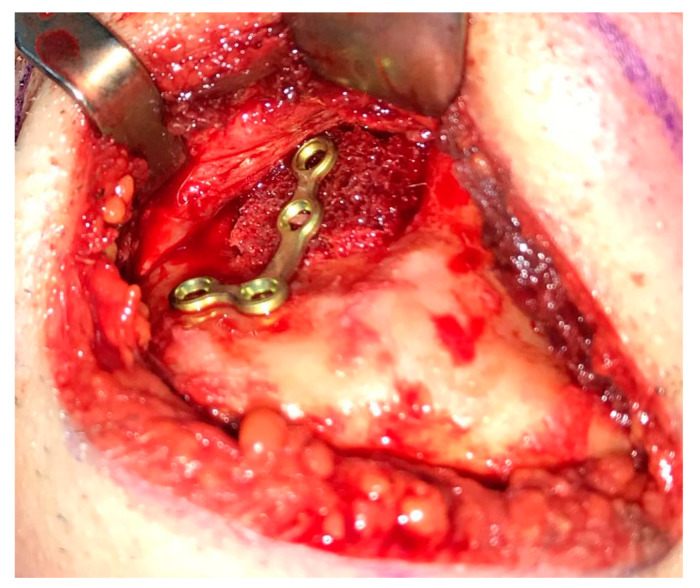
Allogenic bone block fixated to the alveolar ridge using “L” plate with 4 holes, 2 mm diameter and 7 mm length titanium screws.

**Figure 19:**
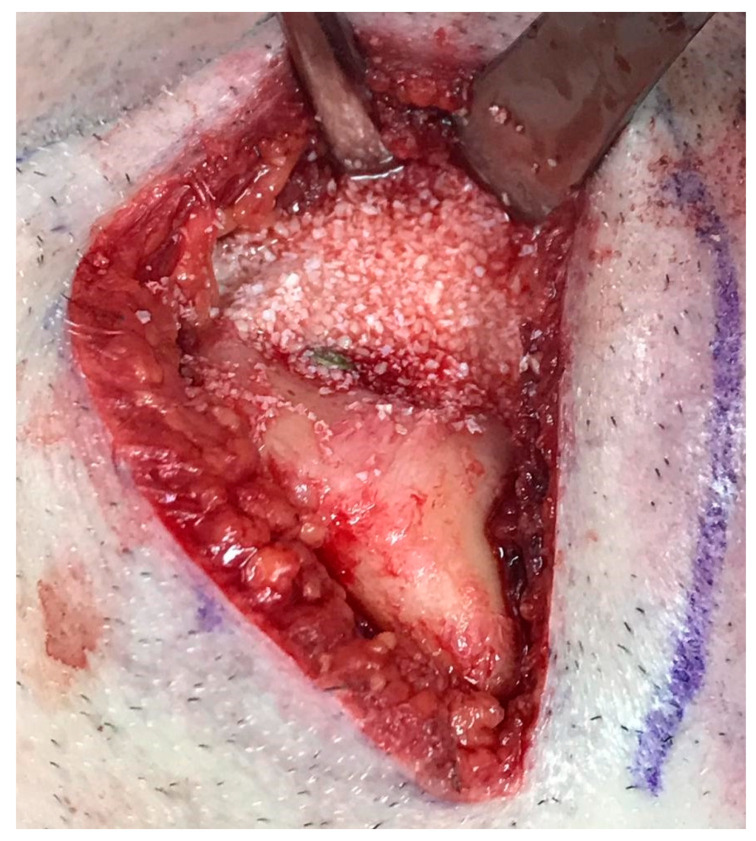
Allogenic bone block covered with xenograft particles.

**Figure 20:**
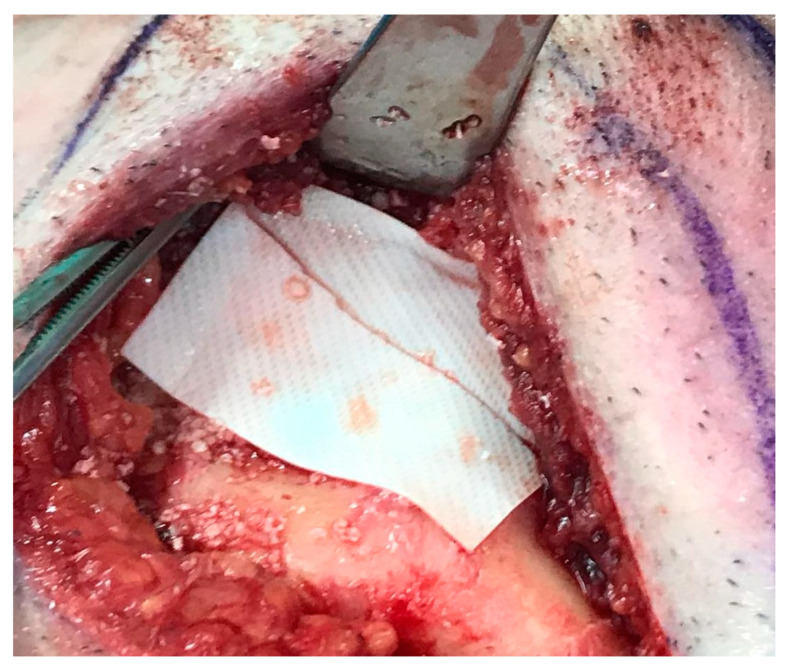
Allogenic bone block covered by cross-linked collagen membrane.

**Figure 21:**
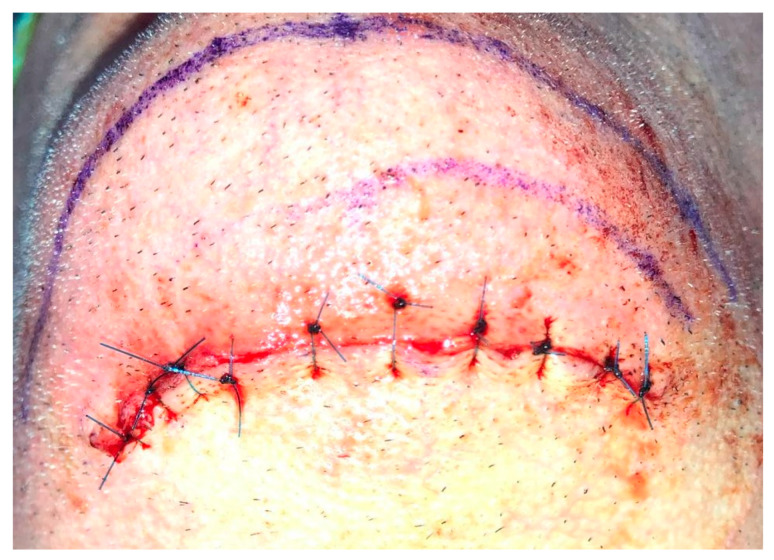
Tissue closure; suturing by layers.

**Figure 22:**
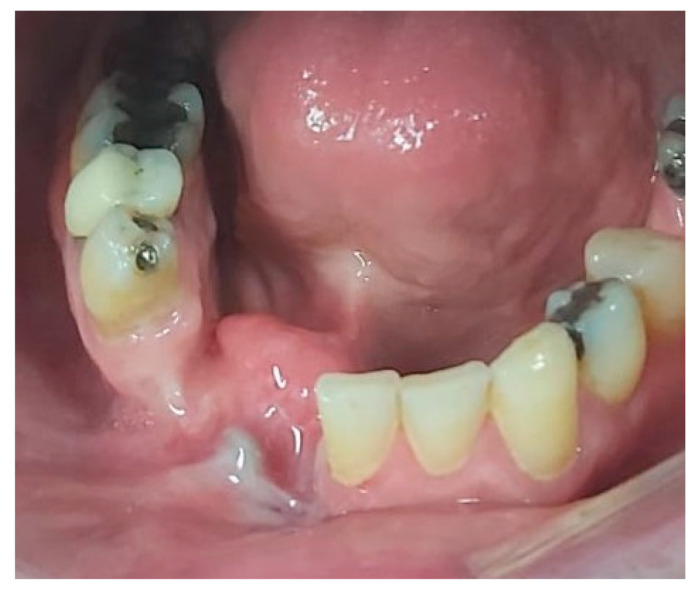
Clinical oral appearance 4 months following reconstruction surgery.

**Figure 23:**
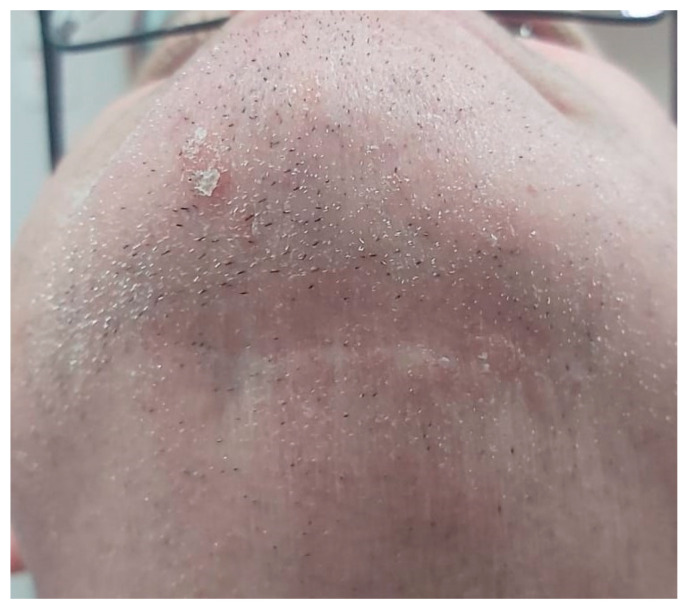
Transcutaneous submental scar 4 months following reconstruction surgery.

**Figure 24:**
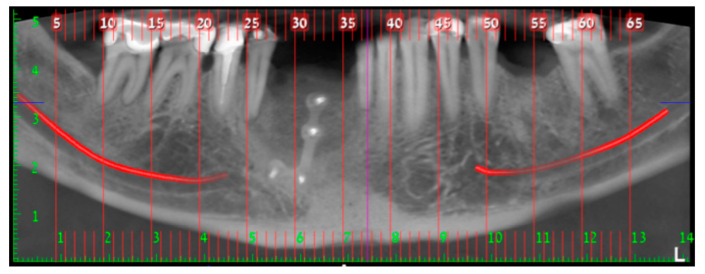
Alveolar ridge bone volume 4 months following reconstruction in CBCT imaging; panoramic image of reconstruction.

**Figure 25:**
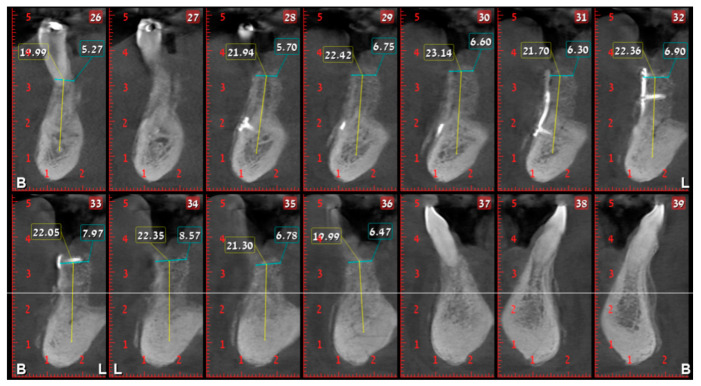
Alveolar ridge bone volume 4 months following reconstruction in CBCT imaging; cross-sections of reconstruction.

**Figure 26:**
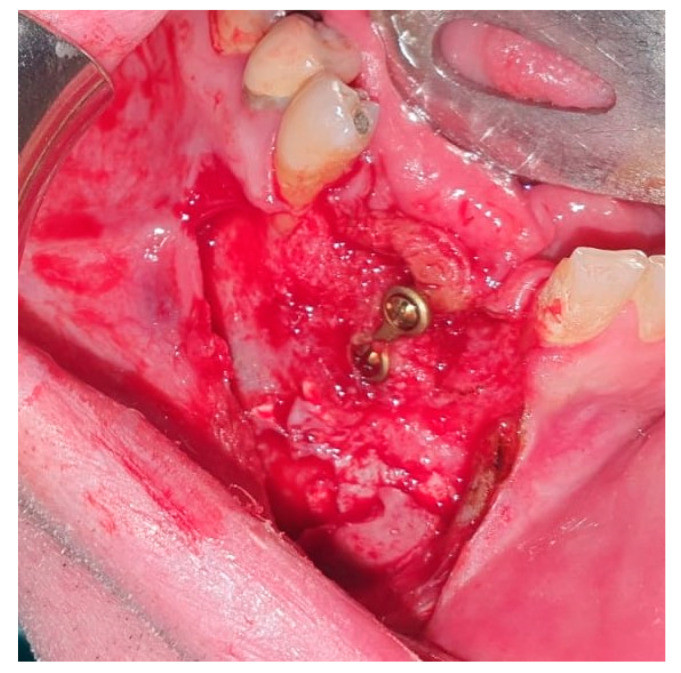
Clinical oral appearance of grafted bone 4 months following reconstruction surgery.

**Figure 27:**
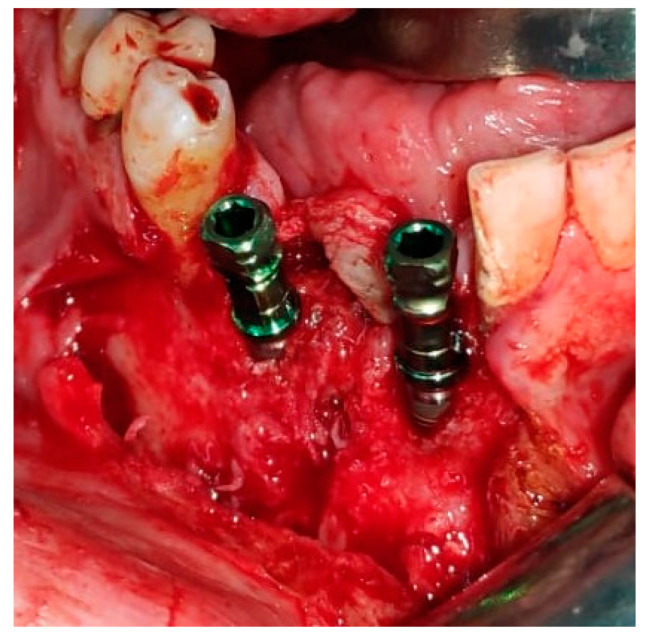
Two dental implants insertion through grafted bone.

**Figure 28:**
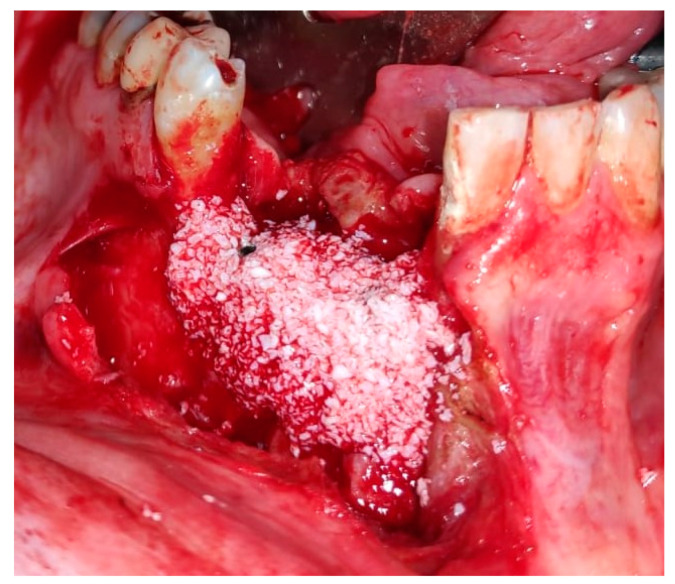
Xenograft particles covering dental implants.

**Figure 29:**
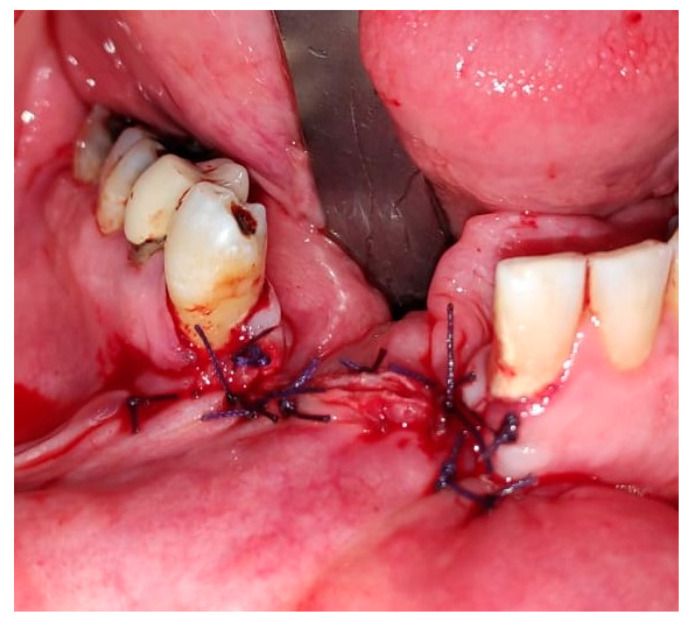
Oral tissue closure; suturing by 3-0 Vicryl.

Reconstruction surgery was conducted by a submental transcutaneous “tent pole” approach in order to preserve oral soft tissue in the defect bed, maintain complete periosteum, and to separate the graft from oral cavity bacteria (Figure 15). The bony defect was exposed (Figure 16) and an allogenic bone block was fitted (Figure 17) and fixated by 2 mm thick titanium plate and screws (Figure 18). The block was covered with xenograft particles and cross-linked collagen membrane (Figure 19 and Figure 20), and the soft tissue was sutured in layers (Figure 21). Four months following reconstruction surgery, the plate was reflected intraorally (Figure 22), and extraorally minimal submental scar was apparent (Figure 23). Bone gain was evident by CBCT (Figure 24 and Figure 25). Two dental implants inserted through the grafted bone, were covered by xenograft particles and cross-linked collagen membrane (Figure 26, Figure 27, Figure 28 and Figure 29).

Total admission days were nine, three days for each operation—resection, reconstruction and implants insertion. Medications prescribed post-operatively were penicillin-based antibiotics and painkillers such as paracetamol and dipyrone. No complications, such as bleeding, pus secretion, local heat and redness, were observed during admission days or during follow-ups.

## 3. Discussion

Accounting for 11% of all odontogenic tumors in the jaw, ameloblastomas affect mostly the ascending ramus and molar region of the mandible [1,2]. According to the World Health Organization (WHO) histopathological classification, there are seven types of ameloblastoma: plexiform, follicular, acanthomatous, granular cell, desmoplastic, peripheral and unicystic [2]. All ameloblastomas, excluding the latter two, have invasive diffuse borders and high recurrence rates, thus the clinical concept of 1 to 3 cm safety margin is needed, even though it is a benign tumor [1,3]. In the case presented, 1 cm of safety bony margin was chosen (Figure 8, Figure 9 and Figure 10).

Reconstruction of bony defects after resection of ameloblastoma remains a major concern for both patients and clinicians [10]. Evaluating the host and classifying of the bony defect can guide the surgeon to patient-specific reconstructive options. The defect involved in the case presented is a two-wall defect, 2 cm vertical and 2 cm latero-lateral sized. Acceptable reconstruction options were distraction osteogenesis (DO), osseocutaneous microvascular free flaps, autogenic bone graft, guided bone regeneration (GBR), and allogenic bone graft. According to a recent systematic review and meta-analyses, if more than 4 mm vertical ridge augmentation is needed, as in the case presented, the GBR technique is less effective and predictable [11]. The presented defect was defined as under the critical size according to Pogrel et al., and thus, we were able to avoid the second and third options, sidestep high resorption rate, and high morbidity following harvesting [12]. DO, involving an osteotomy and gradual elongation of bone using an intra oral device is based on the “tension-stress” principle described by Ilizarov, and can be a good treatment solution, suitable to the amount of bone available in the case (more than 8 mm vertical anterior mandibular bone) [13]. However, it poses high rates of complications; minor, moderate and severe in 58%, 8%, 3% of patients respectively [14].

A good prognostic factor in the case presented is the remaining of vital bone source, since the patient had benign pathology and has not received chemo- or radio-therapy following resection. Vital remaining bone source is able to secrete growth factors and hasten the blood supply for the non-vascularized grafted bone. The clinical and radiographical results of this case, according to all factors discussed, emphasized that the defect presented could be suitably reconstructed by non-vascularized bone graft.

Recombinant human bone morphogenetic protein (rhBMP) is a genetically engineered version of the cytokine that is chemotactic for mesenchymal stem cells and induces their differentiation into osteoblasts [6]. Due to its osteogenic properties, rhBMP has shown its predictability and efficiency. The main function is signaling for regeneration, which promotes differentiation of the stem cells and migrating osteogenic cells [15,16]. Other important factors included in tissue engineering are the scaffolding that is provided by allogenic bone which acts as a framework for bone regeneration, and regenerative cells that are provided by bone marrow aspirate concentrate (BMAC) [17].

A main regenerative concept in bone regeneration is the use of mesenchymal stem cells (MSCs). The oral cavity is populated by MSCs that can be isolated in an easy way and with minimal invasive procedures [18]. Human periapical cyst mesenchymal stem cells (hPCy-MSCs) have features comparable to other dental-derived MSCs. A recent review study by Tatullo et al. [19] stated that this cell population exhibits valuable potentialities that could be of high impact in future regenerative medicine applications, as presented in this case. Recent reports have demonstrated the ability of MSCs to be activated by signals from injured tissues. In these damaged areas, MSCs showed regenerative behavior, promotion of tissue healing, paracrine activities, and secretion of anti-inflammatory factors [20]. A recent special issue, edited by Ballini et al. [21], addressed MSCs in “their different but fundamental roles as promoters, enhancers, and playmakers of the translational regenerative medicine.”

In the case presented, the allogeneic block bone graft serves as a resorbable scaffold. The cancellous portion of the allogeneic block is porous and allows more rapid revascularization and cellular ingrowth. The combination of rhBMP and cancellous allogeneic block, as chosen in the presented case, can complete the “tissue engineering triad” (cells, signaling molecules and scaffold) and more closely matches the properties of autogenous bone [6].

The stress on the chosen scaffold—allogeneic block bone, was not evaluated. Future research can evaluate stress on biomaterials that can be used as a scaffold in bone regeneration, such as the “three-points bending test.” The test, as described by Merrelli et al. [22], may be used to “evaluate these materials’ tolerance to biomechanical forces exhibited by oral cavity tissues.”

To improve the survival rate of the grafted bone, the reconstruction surgery was done via the transcutaneous submental “tent pole” approach, presented by Marx et al. [10]. The approach preserves oral soft tissue in the defect bed and separates the grafted bone from oral cavity bacteria. An additional crucial advantage is keeping the periosteum complete and vital throughout the whole process. Adult human periosteum is highly vascular, retains the ability to differentiate into diverse cell types and accelerates new bone formation underneath it [23]. The success of the bone graft and dental implants highly depends on the blood supply coming from the host tissue. Recent studies by Isola et al. emphasized the importance of vitamin D and malondialdehyde in this complex process [24,25]. One of the main functions of vitamin D is in the regulation of serum calcium levels, alveolar bone growth and periodontal ligament homeostasis [26].

Extraoral esthetics achieved using the approach are high (Figure 23), and the scar is minimal and almost invisible in natural head position [27]. Placement of the submental incision posterior to the submental crease, as done in the case presented (Figure 15 and Figure 16), will result in an inconspicuous, well-concealed scar [27]. Roh examined clinical outcomes of the submental approach, compared with a conventional submandibular approach, for submandibular gland resection and concluded that the scar results in the former were usually less visible and the patient satisfaction score was higher [27].

## 4. Conclusions

Mandible reconstruction modalities have a crucial influence on patient quality of life, function and esthetics. This case report presents an alternative treatment in which an allogenic bone block combined with rhBMP and xenograft particles can replace the traditional autogenous bone in specific cases. The submental transcutaneous “tent pole” approach can preserve oral soft tissue in the defect bed, maintain complete periosteum coverage and separate the graft from oral cavity bacteria. In this way it can improve the success rate of the reconstruction procedure. For future studies, important factors such as vitamin D and malondialdehyde should be monitored as they have an impact on graft and dental implant success [24,25].
